# Combinatorial Mismatch Scan (CMS) for loci associated with dementia in the Amish

**DOI:** 10.1186/1471-2350-7-19

**Published:** 2006-03-03

**Authors:** Jacob L McCauley, Daniel W Hahs, Lan Jiang, William K Scott, Kathleen A Welsh-Bohmer, Charles E Jackson, Jeffery M Vance, Margaret A Pericak-Vance, Jonathan L Haines

**Affiliations:** 1 Center for Human Genetics Research and Department of Molecular Physiology and Biophysics, Vanderbilt University Medical Center, Nashville, TN, USA; 2 Center for Human Genetics and Department of Medicine, Duke University Medical Center, Durham, NC, USA; 3 Joseph & Kathleen Bryan ADRC/Division of Neurology, Duke University Medical Center, Durham, NC, USA; 4 Scott & White, Temple, TX, USA

## Abstract

**Background:**

Population heterogeneity may be a significant confounding factor hampering detection and verification of late onset Alzheimer's disease (LOAD) susceptibility genes. The Amish communities located in Indiana and Ohio are relatively isolated populations that may have increased power to detect disease susceptibility genes.

**Methods:**

We recently performed a genome scan of dementia in this population that detected several potential loci. However, analyses of these data are complicated by the highly consanguineous nature of these Amish pedigrees. Therefore we applied the Combinatorial Mismatch Scanning (CMS) method that compares identity by state (IBS) (under the presumption of identity by descent (IBD)) sharing in distantly related individuals from such populations where standard linkage and association analyses are difficult to implement. CMS compares allele sharing between individuals in affected and unaffected groups from founder populations. Comparisons between cases and controls were done using two Fisher's exact tests, one testing for excess in IBS allele frequency and the other testing for excess in IBS genotype frequency for 407 microsatellite markers.

**Results:**

In all, 13 dementia cases and 14 normal controls were identified who were not related at least through the grandparental generation. The examination of allele frequencies identified 24 markers (6%) nominally (p ≤ 0.05) associated with dementia; the most interesting (empiric p ≤ 0.005) markers were D3S1262, D5S211, and D19S1165. The examination of genotype frequencies identified 21 markers (5%) nominally (p ≤ 0.05) associated with dementia; the most significant markers were both located on chromosome 5 (D5S1480 and D5S211). Notably, one of these markers (D5S211) demonstrated differences (empiric p ≤ 0.005) under both tests.

**Conclusion:**

Our results provide the initial groundwork for identifying genes involved in late-onset Alzheimer's disease within the Amish community. Genes identified within this isolated population will likely play a role in a subset of late-onset AD cases across more general populations. Regions highlighted by markers demonstrating suggestive allelic and/or genotypic differences will be the focus of more detailed examination to characterize their involvement in dementia.

## Background

With over 4 million individuals affected with Alzheimer's disease (AD) in the U.S., dementia of the Alzheimer's Type (DAT) is the leading cause of dementia in the elderly. These current estimates are projected to triple over the next 50 years as the population ages [1-3]. AD has a complex etiology with strong genetic and environmental determinants. Tremendous evidence suggests the involvement of at least three genes in early-onset autosomal dominant AD. Amyloid precursor protein (APP on chromosome 21) [4,5], presenilin I (*PSEN1 *on chromosome 14) [6-9], and presenilin II (*PSEN2 *on chromosome 1) [10,11] are all prominent early-onset Alzheimer's disease genes. Understanding of the more common late-onset Alzheimer disease (LOAD), is centered on the role of one universally accepted risk gene, the apolipoprotein E locus (*APOE*) [12]. The *APOE *ε4 allele (frequency approximately 16%) [13,14] acts in a dose-related manner to increase risk for LOAD and decrease age-of-onset [15,16]. Although its involvement is without question, *APOE *accounts for less than half of late-onset AD susceptibility [15]. Given the strong heritability of AD, other genetic factors are likely to be involved. Multiple linkage screens have been conducted to elucidate additional regions harboring susceptibility genes for late-onset AD [17-35]. While regions on chromosomes 9, 10 and 12 are most consistently identified, candidate genes within those regions have yet to be clearly implicated in AD. Meanwhile, numerous other regions have been implicated but have not been the focus of detailed study due to the prominence of 9, 10, and 12.

Though numerous promising LOAD candidate genes have been examined, the lack of replication across studies has made a definitive declaration of their involvement difficult (Reviewed in [36,37]). Genetic heterogeneity is likely to be one of the underlying reasons for this lack of replication. Given this, one possible solution is to study populations likely to be more genetically homogeneous, thereby enriching for a more homogeneous set of risk alleles. The North American Amish population is a relatively isolated, genetically well-defined homogeneous population, well-suited for this type of study. Further detail regarding the establishment of the North American Amish population has been described elsewhere [38-43]. While there may be a number of LOAD susceptibility genes contributing to disease in the general population, the relatively homogeneous Amish population is likely to contain a smaller set of risk alleles.

One challenge in performing linkage analysis in Amish pedigrees is to utilize the extensive pedigree information available while maintaining tractability of the computations. Due to their strong religious and cultural beliefs, the Amish very rarely marry outside of their communities, thereby promoting a genetically isolated population [38-41]. This in turn has led to an elevated degree of consanguinity, yielding family pedigrees that contain many loops that can often be traced back three or more generations. In fact, through use of the Anabaptist Genealogy Database (AGDB), we find that 93% of our overall study population of 460 individuals and more specifically 25/27 (93%) of the individuals used in this study can be traced back 10 generations to a single founding couple. Moreover, 100% of individuals within our entire Amish sample (460) belong to one very large extended pedigree when allowing parent-child and marriage links to be included [44]. Since LOAD cannot be ascertained until late in life, affected individuals are usually only available for genotyping in a single generation. Hence, by far, most of the individuals in the pedigree have unknown phenotype and genotype status. It should be noted that there are limited methodologies available to analyze disease gene linkage utilizing such large complex pedigrees. One such method is SimWalk2 which utilizes descent graph theory and Markov Chain Monte Carlo (MCMC) simulation to compute lod scores [45]. This is a computationally demanding process and because of the uncertainty of MCMC convergence, the accuracy of the scores obtained may be difficult to assess.

Combinatorial mismatch scanning (CMS) is an alternative technique to search for IBS sharing in distantly related individuals from isolated founder populations where standard linkage and association analyses are difficult to implement. While several other methods could be implemented, this approach was used because of its simplicity in examining existing data. This method was also chosen because at the onset of analysis, we lacked the more detailed knowledge of the inter-relatedness of our sample often required to perform similar, but more sophisticated approaches within large inbred pedigrees [46-48]. This strategy is designed to circumvent the confounding issue of genetic heterogeneity, by examining affected and unaffected persons from relatively small founder populations [49]. By genetically evaluating case and control individuals selected from such a population, whose common ancestor is no more closely related than grandparents, some prevailing problems in allelic association studies of complex disease within generally outbred populations can be avoided. Population stratification can lead to allelic association and be misinterpreted as linkage disequilibrium. In this approach, population stratification is less of an issue due to the relative isolation and common heritage of the study population. Another difficulty facing genetic studies within large outbred populations is that these populations are likely to exhibit locus heterogeneity. Within an isolated population, the probability that the risk allele of interest might have entered the gene pool only once or rarely, provides a great advantage. This in turn will likely facilitate the distinction between true and spurious association. Heath and colleagues highlight another advantage of examining isolated populations by alluding to the potential for detecting gene-gene interactions [49]. These epistatic interactions may play a substantial role in complex disease, effectively hampering the ability to detect association using single locus methods within heterogeneous populations. With reference to this problem, there are good reasons to believe that epistatic (gene-gene) interactions are ubiquitous in complex disease and may in fact be more important than single-gene effects [50].

## Methods

### Subjects and phenotypes

The Amish often have large sibships and extensive pedigree records that permit the accurate estimation of IBS gene sharing to be accurately evaluated. The estimated coefficient of inbreeding for the entire population is 0.0151, which is approximately equivalent to having second cousins as parents [51]. This effect has led present-day Amish to possess genes inherited identically from a common ancestor at rates higher than observed in the general population. By searching the Anabaptist Genealogy Database (AGDB) with the query software PedHunter, we have determined the level of relatedness of our sample more precisely [44,52]. We calculated the average kinship coefficient for our overall ascertained Amish sample to be 0.019 ± 0.00053 (mean ± SEM). This measure demonstrates a significant difference from the average kinship coefficients calculated for the within cases group (0.011 ± 0.0013, mean ± SEM), the within controls group (0.0094 ± 0.0011, mean ± SEM), and the between cases and controls group (0.010 ± 0.00065, mean ± SEM). These calculations provide us with additional confidence that our cases and controls are more distantly related to each other relative to our overall sample population.

The subjects included in this study are a subset of individuals described in extensive detail elsewhere [43]. Briefly, individuals enrolled in the study each were assigned to one of three clinical impression categories; dementia (probable or possible Alzheimer's disease); unclear (includes mild cognitive impairment (MCI)); or unaffected (cognitively normal). Participants were administered the Mini-Mental State Exam (MMSE) [53], with possible scores ranging from 0 to 30. All individuals scoring 27 or greater were classified as cognitively normal/unaffected. Those scoring 23 or less were classified as cognitively impaired and labeled as probable dementia. Those who scored 24–26 had additional neuropsychological testing including the Dementia Rating Scale (DRS) [54], the Boston Naming Test (BNT) [55], and a reading subtest from the Wide Range Achievement Test-Revised (WRAT-R) [56]. Persons were categorized as having mild cognitive impairment if their DRS score fell below an age-adjusted threshold. Each case was discussed and a consensus "final" diagnosis was determined using all available information. For analytical purposes, the cases were classified as affected (demented), unclear (includes MCI), and unaffected (cognitively normal).

Five Amish pedigrees were included in this study. Three families were from Elkhart and LaGrange counties in Indiana, one extended family from Adams county Indiana, and one extended family from Holmes county Ohio. The extended pedigree from Adams county has been the subject of other previous and ongoing studies of dementia in the Amish [16,57]. Among the 115 individuals who were genotyped, 40 were classified as having dementia, 9 were classified as unclear, and 66 individuals were unaffected. To minimize chance IBS inheritance, individuals selected for the CMS analysis were unrelated through the grandparental generation [49]. For this current study we identified 13 dementia cases and 14 cognitively normal individuals who met this requirement. This study was undertaken after Institutional Review Board review and approval.

### Molecular analysis

Following informed consent, blood samples were collected from each individual and genomic DNA was extracted from blood using standard procedures. Cell lines have been initiated on most sampled individuals. All DNA samples were coded and stored at 4°C until used.

Markers were genotyped at both the Vanderbilt and Duke laboratories for all DNA samples. Laboratory personnel were blinded to pedigree structure, affection status, and location of quality control samples. Duplicate quality control samples were placed both within and across plates and equivalent genotypes were required for all quality control samples to ensure accurate genotyping. At the Vanderbilt laboratory, marker primer sequences were obtained from the Genome Database [58] or designed with Primer3 software [59] and synthesized by Invitrogen Life Technologies (Carlsbad, CA). Amplification was performed in a PCR Express machine (ThermoHybaid, Needham Heights, MA) with the following conditions: 94°C-4 min.; 94°C-15 sec., AT-30 sec., 72°C-45 sec. (35 cycles); 72°C-4 min. PCR products were denatured for 3 min. at 95°C and run on a 6% polyacrylamide gel (Sequagel-6^®^from National Diagnostics, Atlanta, GA) for ~1 hr. at 75 W. Gels were stained with a SybrGold^®^rinse (Molecular Probes, Eugene, OR) and scanned with the Hitachi Biosystems FMBIOII laser scanner (Brisbane, CA). Marker genotyping at the Duke laboratory was performed using fluorescence imaging (Molecular Dynamics SI Fluorimager) and a semi-automated allele calling system [60].

Hardy-Weinberg equilibrium calculations were performed for each marker and Mendelian inconsistencies were identified using PedCheck in the overall dataset [61]. Suspect genotypes were re-read by a different technician or re-run as necessary to reduce errors. All microsatellite markers were required to have >90% of possible genotypes to be included in the analysis.

### Statistical analysis

Comparisons between case and control genotype data for the 407 microsatellite markers were conducted using the R software package to perform Fisher's exact tests in r × c Contingency Tables [62-64]. Each marker was examined for both allele and genotype differences between individuals affected with dementia and those without dementia. The first test was for IBS allele frequency inequality between the two classes, and the second test was for IBS genotype frequency inequality between the two classes. Fisher's exact test computes the probability *p *that the pattern of alleles observed in the sample would be obtained if there were truly no difference between the allele frequencies among affected and unaffected individuals. While our current sample size is adequate to detect moderate to major effects (odds ratio of >6 with 80% power), it does not preclude our ability to detect smaller effects given that these power calculations are based on the assumptions of complete independence of samples and random sampling of the population, neither of which is true.

To empirically evaluate the statistical significance of the p-values computed in the CMS study, we permuted our dataset. We randomly re-assigned affection status for each of the 27 individuals maintaining the original total of 13 cases and 14 controls. We then executed the Fisher's exact test using the same allele and genotype data in the original dataset for each of the 407 markers. The distribution of p-values obtained from Fisher's exact testing on 1000 randomized sets of data was then created for both the allele and genotype comparisons to assess the empiric thresholds. We would expect the Fisher's exact p-value to match the p-value within the large distribution. These permutations were needed to correct for any residual bias from unrecognized kinship correlation present.

## Results

We tested 407 microsatellite markers for differences in both allele frequency and genotype frequency between Amish dementia cases and controls. We considered all pointwise p-values and have chosen to report only markers demonstrating Fisher's exact p-values < 0.05 for either allele or genotype frequency differences. This arbitrary threshold was chosen to limit the results to be displayed and to provide a reference point for discussion of markers demonstrating nominally significant (albeit within the null expectation given the number of markers examined) evidence of association to dementia within our population.

As an example, Table 1a shows the allele count data for marker D5S211. There are eight D5S211 alleles in the sample with 27 subjects being typed for 54 alleles. In the example, the probability of this data being obtained if there were no underlying difference between the allele distributions for the two classes is < 0.005 (Table 2). Table 1b shows the genotype data for marker D5S211. Note that out of the thirteen genotypes observed in the data only one genotype is present in both affected and unaffected classes. The probability of the data being obtained if there were no underlying difference between the genotype distributions for the case and controls is < 0.005 (Table 2). Markers demonstrating nominally significant (p ≤ 0.05) differences between cases and controls are listed in Table 2. There were 24 out of 407 markers (6%) demonstrating significant differences in allele frequency. The most significant markers were D3S1262, D5S211, and D19S1165. When examining the markers for genotype frequency differences, 21 out of 407 markers (5%) were significantly different between our dementia cases and controls. The most significant markers were D5S1480 and D5S211. While there were seven markers (D3S1262, D4S1625, D5S211, D6S1031, D8S1477, D8S272, D17S921, and D18S481) with p-values ≤ 0.05 for both the allelic and genotypic tests, only one marker (D5S211) was significant at the empiric p ≤ 0.005 level for both tests. Although all findings are uncorrected and there are no findings with genome-wide significance, markers in close proximity to those regions previously identified are of particular interest for future study.

**Table 1:** Comparison of Allele and Genotype frequencies for D5S211 in dementia cases and controls

Allele counts			
Alleles	Cases	Controls	Totals

186	2	0	2
192	4	8	12
196	2	4	6
198	1	1	2
**200**	**16**	**4**	20
206	1	1	2
202	0	6	6
204	0	4	4

Totals	26	28	54

Genotype counts			

Genotypes	Cases	Controls	Totals

186/200	2	0	2
192/192	0	3	3
192/196	0	1	1
192/200	4	0	4
192/206	0	1	1
196/198	0	1	1
196/200	2	1	3
196/202	0	1	1
198/206	1	0	1
200/200	4	0	4
200/202	0	3	3
200/204	0	2	2
204/204	0	1	1

Totals	13	14	27

Bold highlights allele demonstrating greatest difference

**Table 2:** Microsatellite markers demonstrating nominally significant (p ≤ 0.05) empiric p-values for allele and genotype frequency differences between dementia cases and controls. Microsatellite markers in close proximity to those demonstrating significance in this study and found to be either linked (lod ≥ 1) or associated (p ≤ 0.05) in previous studies are also listed.

Chromosome	Map Position (cM)	Mb Location	Marker	Fisher's Exact p-value		Empiric p-value		Max Lod Score	Study
				Allele	Genotype	Allele	Genotype		
1	25	11.4	D1S2667	0.162	0.007	0.170	0.015		
1	64	32.1	D1S396	0.043	0.449	0.050	0.407		
2	38	17.4	D2S1360	0.028	0.243	0.035	0.228		
2	74	50.7	D2S1352	0.200	0.026	0.208	0.032		
2	252	237.9	D2S2968	0.688	0.018	0.684	0.025		
3	119	103.7	D3S2459	0.223	0.007	0.231	0.014		
3	153	140.7	D3S1764	0.029	0.271	0.035	0.248		
3	177	168.7	D3S1763					1.69	Hahs et al.
3	201	187.5	D3S1602			0.007**			Hiltunen et al.
3	201	187.7	***D3S1262***	**0.001**	**0.019**	***0.003***	**0.026**		
3	209	191	D3S2398					2.16	Hahs et al.
3	216	193.8	D3S2418					1.18	Hahs et al.
4	78		D4S2367	0.557	0.015	0.557	0.022		
4	130	130.7	D4S2394					2.12	Hahs et al.
4	146	143.9	**D4S1625**	**0.032**	**0.013**	**0.038**	**0.020**		
4	154	152.5	D4S1548					3.01	Hahs et al.
4	158	158.7	D4S1629					1.32	Pericak-Vance et al. (2000)
5	8		D5S2849	0.590	0.031	0.589	0.038		
5	92	82.3	D5S1347	0.060	0.002	0.068	0.007		
5	98	89.2	D5S1725					1.47	Hahs et al.
5	147	144.1	*D5S1480*	0.465	0.001	0.467	*0.005*		
5	175	168.4	D5S400	0.04*					Farrer et al.
5	183	173.2	***D5S211***	**0.001**	**0.001**	***0.002***	***0.004***		
5	183	173.2	D5S211					1.3	Blacker et al.
6	89	77.5	D6S1031	0.024	0.046	0.030	0.051		
6	160	158	D6S1007	0.933	0.017	0.923	0.025		
8	60	32.2	**D8S1477**	**0.004**	**0.018**	**0.007**	**0.026**		
8	125	118.5	D8S592	0.387	0.032	0.391	0.038		
8	154	137.8	**D8S272**	**0.007**	**0.021**	**0.010**	**0.028**		
9	14		D9S2169	0.022	0.394	0.027	0.362		
10	63	35.3	D10S1208	0.013	0.247	0.018	0.231		
10	76	57.2	D10S1221	0.028	0.054	0.034	0.059		
12	78	66.2	D12S1294	0.220	0.045	0.228	0.050		
13	39	42.1	D13S325	0.027	0.070	0.033	0.072		
13	76	96.7	D13S892	0.040	0.224	0.047	0.210		
14	44	37.4	D14S306	0.020	0.104	0.026	0.103		
14	94	86.3	D14S612	0.016	0.166	0.021	0.156		
15	101	92.8	D15S816	0.046	0.158	0.053	0.150		
15	116	98.9	D15S87	0.031	0.083	0.037	0.084		
16	64	49.7	D16S3396	0.039	0.450	0.046	0.409		
16	130		D16S2621	0.227	0.036	0.235	0.042		
17	36	14.2	**D17S921**	**0.024**	**0.026**	**0.030**	**0.032**		
17	126	77.8	D17S928	0.024	0.201	0.029	0.186		
18	7	3.1	**D18S481**	**0.017**	**0.006**	**0.022**	**0.013**		
18	109		D18S1362	0.431	0.021	0.434	0.028		
19	21	6.1	D19S1034			0.013**			Hiltunen et al.
19	33	9.7	D19S586					2.06	Hahs et al.
19	36	12.2	*D19S1165*	0.002	0.066	*0.004*	0.069		
20	39	17.3	D20S470	0.027	0.206	0.033	0.191		
21	27	30.6	D21S1270	0.245	0.010	0.253	0.018		

Bold denotes markers nominally significant (p ≤ 0.05) in both allele and genotype comparisions

Italics highlights markers empirically significant at p ≤ 0.005

*SAS software was used to measure significant differences in allele frequency between DAT cases and controls

**Pearson's chi-square was calculated and then empirical significance was determined through examination of 1000 replicated datasets.

## Discussion

We have detected a few microsatellite markers of particular interest, which demonstrate significant differences between dementia cases and controls within our Amish founder population using the combinatorial mismatch scanning approach. The CMS concept is based on excess IBS allele/genotype sharing between individuals sharing a distant set of common founders [49]. The most noteworthy finding is on chromosome 5q35.2 at approximately 183 cM where we find evidence for both allele and genotype differences between our dementia cases and controls for marker D5S211. In their large genome-wide linkage study of Alzheimer's disease, Blacker et al. detected a multipoint lod score of 1.3 at this same marker [32]. In a recent study of consanguineous Israeli-Arab communities, Farrer and colleagues found significant evidence for allele frequency differences between AD cases and controls at the closest marker (D5S400 at 175 cM) on chromosome 5 run in their study [33]. Positive findings across three distinct study populations suggest that a gene or genes within this region of chromosome 5 may be involved in risk for dementia of the Alzheimer's type (DAT). Thus future examination of this region on chromosome 5 within our Amish families is warranted. While another marker on chromosome 5q31.3 (D5S1480 at 147 cM) demonstrated genotype differences between dementia cases and controls, this location is novel with respect to other previous studies.

We also found evidence of allele frequency differences on chromosome 3q27.3 at D3S1262 (201 cM). One study tested for association with AD in a geographically distinct Finnish population descended from a small group of original founders [65]. This group found significant association (empiric p = 0.007) at marker D3S1602 (also located at 201 cM) within their AD sample. An interesting candidate gene at this location is *SST*, the gene encoding somatostatin, which functions as a neurotransmitter in the central nervous system. Somatostatin inhibits the release of glucagon, growth hormone, gastrin, insulin, and secretin. Additional evidence for this region stems from our genome-wide linkage study within the Amish population, for which we observed a suggestive two-point lod score of 2.42 at the nearby marker D3S2398 (209 cM) [43].

An additional marker demonstrating suggestive allele frequency differences is located on chromosome 19p13.2 (D19S1165 at 36 cM). Hiltunen et al. had detected evidence for association at two nearby markers (D19S1034 and D19S433) spanning the region containing our significant results [65]. ICAM-1 (Intercellular Adhesion Molecule 1), a previously-reported AD candidate gene, also lies within this region of interest. Pola et al. showed that the ICAM-1 K469E gene polymorphism was associated with AD in an Italian population [66]. This association was not, however, supported in studies of the gene in Finnish and Spanish populations [67,68]. Additional strong evidence from previous work indicates the presence of a late-onset AD locus within this region. A study by Wijsman et al. provides substantial evidence for a locus at approximately 35 cM affecting AD age at onset [69]. While our study does not address age at onset, it further suggests the involvement of this region in AD.

Another region of relative interest is on chromosome 4q31.2 at marker D4S1625. This marker located on chromosome 4q at approximately 146 cM lies between two markers (D4S2394 at 130 cM and D4S1548 at 154 cM) demonstrating highly suggestive evidence for linkage within our Amish population [43]. Further evidence for this region stems from work by Pericak-Vance et al. where they detect modest evidence for linkage to a marker only 4 cM away (D4S1629, lod = 1.32) from D4S1625 (Table 2) [26].

Given that our data may violate assumptions (i.e. normality and/or unrealized correlation) of the Fisher's exact test, we determined the empiric p-value for our results through permutation. We performed the Fisher's exact test on 1000 replicates containing the same original genotype data, but with randomized affection status. The resulting distribution of p-values was then used as an empiric measure of significance for our results (Table 2). On the whole, the empiric p-value thresholds for our study showed the Fisher's exact p-value to be somewhat more liberal than expected.

We have previously performed a genome-wide linkage screen for dementia within this population; however the complex nature of the Amish pedigrees provides a challenge for linkage analysis, given the size and number of consanguineous loops within these extended families. Accordingly the linkage analysis by itself does not allow taking full advantage of the data available to us. To examine our data more thoroughly, we performed the combinatorial mismatch scan. Both this approach and the linkage analysis utilize the high level of inter-relatedness, within the Amish population, to their advantage. The nature of the CMS analysis, allowed us to examine these data without being computationally burdened by the size or family structure of our population. These two methods complement each other by allowing the examination of the same data using both a family-based approach and a "pseudo" case-control approach to identify regions across the genome which are potentially involved in AD susceptibility. We are fully aware of the limited power of our current sample; however, these analyses should be viewed as an adjunct to our recent genomic screen.

## Conclusion

We have reported several markers across the genome (chr3, 4, 5, and 19) to have significant allelic and/or genotypic frequency differences between dementia cases and controls within the combined Amish communities of Ohio and Indiana. While the evidence presented here is not overwhelming for any specific region, these results must be viewed in conjunction with not only our genomic screen but with findings across other studies within additional populations. In conclusion, our results provide the groundwork for future detailed study of these regions within our growing sample of Amish individuals.

## Competing interests

The author(s) declare that they have no competing interests.

## Authors' contributions

JLM directed and performed some of the analyses, collated the results, and was responsible for preparing and editing the manuscript and tables therein. DWH was involved in the drafting of the manuscript and providing input on the analyses. LJ was responsible for data management and analysis. WKS provided input for the analysis, helped in editing the manuscript, and provided financial support through grant funding. KAW was key in all clinical evaluations and provided input for the manuscript. CEJ has been a longtime consultant for ascertainment and recruitment from the Amish community due to his extensive interaction within this isolated population. JMV coordinated the genotyping of the microsatellite markers used within this study. JLH and MPV are Principal Investigator (PI) and co-PI, respectively. Both PIs were instrumental in providing the infrastructure, aiding in the study design, providing input in the manuscript, and supporting this project as well as additional projects surrounding this manuscript through their grant funding.

## Pre-publication history

The pre-publication history for this paper can be accessed here:


